# Primary B-cell Lymphoma of the Thoracic Spine: A Rare Cause of Spinal Cord Compression

**DOI:** 10.7759/cureus.16608

**Published:** 2021-07-24

**Authors:** Zamzuri Zakaria@Mohamad, Ren Yi Kow, Chooi Leng Low, Asmah Hanim Hamdan, Mohd Shukrimi Awang

**Affiliations:** 1 Department of Orthopedics, Traumatology, and Rehabilitation, International Islamic University Malaysia, Kuantan, MYS; 2 Department of Radiology, International Islamic University Malaysia, Kuantan, MYS; 3 Department of Pathology, International Islamic University Malaysia, Kuantan, MYS

**Keywords:** spinal cord compression, primary b-cell lymphoma, thoracic spine, oncology, spinal tuberculosis

## Abstract

Primary non-Hodgkin lymphoma arising from the spine is exceedingly rare. Spinal cord compression can be the first presentation of a patient with primary spinal non-Hodgkin lymphoma. Due to its rarity and vague clinical presentation, the diagnosis can be confused with tuberculosis of the spine, a more common disease in this country. We present a case of primary thoracic spine B-cell lymphoma in a 45-year-old lady who presented with spinal cord compression. This case highlights the importance of obtaining histopathological samples for examination and the treating physician should be vigilant on this rare cause of spinal cord compression. Treatment can be initiated promptly once the diagnosis is established as primary spinal non-Hodgkin lymphoma carries a dire prognosis.

## Introduction

Malignant lymphoma is a type of malignancy derived from the lymphoid tissue, and it normally presents as lymphadenopathy or a solid tumor [1,2]. Compared to Hodgkin’s lymphoma, non-Hodgkin lymphoma (NHL) is much unpredictable and it has a greater predilection to spread to extra-nodal locations [2]. Primary lymphoma affecting the skeletal system is uncommon, with only 1% of NHL arising from the bone [3-6]. In practice, primary NHL originating from the spine is exceedingly rare, accounting for only 0.1% of all NHL, and it may present with spinal cord compression as the first symptom [3-6].

In a country where tuberculosis is endemic, tuberculosis is the first diagnosis that comes to a physician’s mind when he encounters a young patient presenting with unexplained spinal cord compression [7,8]. Coupled with the rarity of spinal involvement of non-Hodgkin lymphoma, this diagnosis can be easily missed. We present a rare case of primary B-cell lymphoma of the thoracic spine causing spinal cord compression in a middle-aged lady. 

## Case presentation

A 45-year-old lady with no known medical illness presented with progressive bilateral lower limb weakness and numbness for five months. Symptoms started insidiously and gradually worsened prior to presentation. There was associated occasional shortness of breath as well as bladder and bowel incontinence one week prior to presentation. There was no neck or back pain. She had loss of appetite and loss of weight of non-specific duration. She had no fever, night pain, abnormal neck, breast, or abdominal swelling. She denied history of trauma or falls.

Clinically, she was bed-bound and appeared cachexic. Spine examination revealed mild tenderness at the mid-thoracic spine with no step deformity. Neurologically, both of her lower limbs had motor deficit with power of Medical Research Council (MRC) grade 0 and absent sensation from T5 downwards. The muscle tone was hypertonic and lower limb reflexes were brisk. Her anal tone was also lax with loss of perianal sensation. Otherwise, her upper limb neurology was normal. There was no palpable breast lump, thyroid nodule, or mass per abdomen. There was no enlarged lymph node at the cervical, axillary, or inguinal area. Lung examination revealed reduced air entry at the right side.

**Figure 1:**
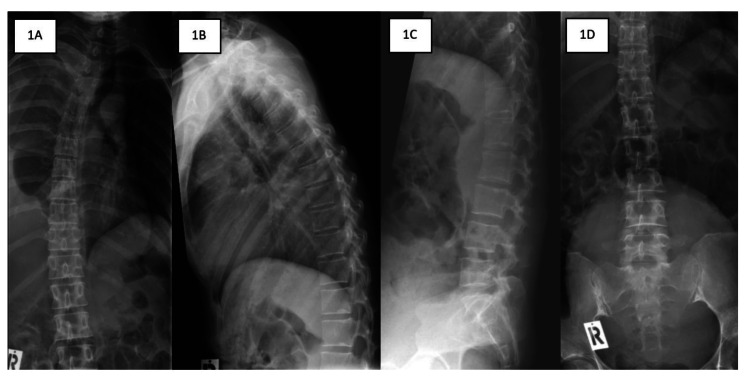
The plain radiographs of the thoracic and lumbar spine revealed no gross abnormality.

**Figure 2:**
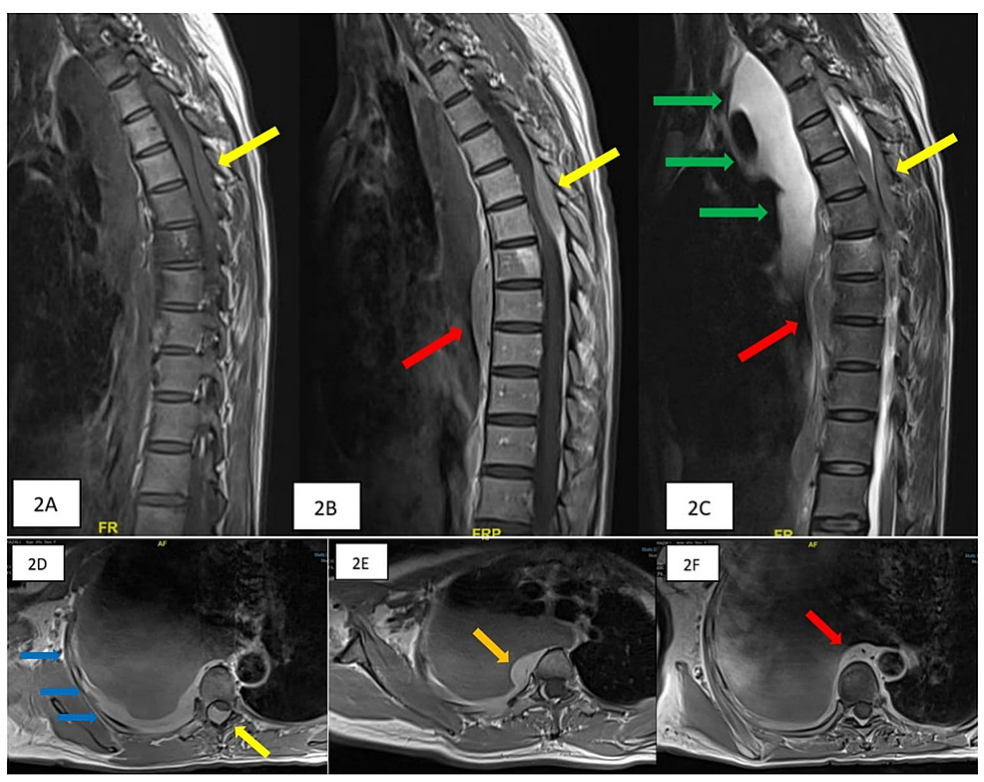
Magnetic resonance images of the thoracic spine. The sagittal plane (A-C) shows an intraspinal extradural lesion extending from T4 to T7 (yellow arrow), measuring about 6 cm in length. It is slightly hyperintense to the spinal cord on T1WI (A) and T2WI (C) and shows marked enhancement on T1 post-contrast images (B). (D-F) T1 axial post-contrast images. Abnormal signal intensity and contrast enhancement are also seen at the T7 vertebral body (A-C). The extradural mass has compressed and displaced the thecal sac left anterolaterally (D) with total obliteration of the subarachnoid space at the level of T6. Homogeneously enhancing prevertebral (red arrow) (A-C, F at the level of T7-T9) and right paravertebral lesions (orange arrow) (E, at the level of T2-T9) are also noted. There is also thickening of the right pleura (blue arrows) (D) with right pleural effusion (green arrows).

Biochemically, her white cell count was normal (7.1 x 10^9^/L) with lymphocyte of 13.8%. The erythrocyte sedimentation rate (ESR) was 24 mm/h with C-reactive protein level of <0.05 mg/dL and the serum lactate dehydrogenase (LDH) level was 297 U/L. Other blood parameters such as renal profile and liver function test were normal. Mantoux test was equivocal, and the sputum acid-fast bacilli test was negative. Plain chest radiographs showed right pleural effusion until mid-zone. No abnormality detected on plain radiographs of the thoracolumbar spine (Figures 1A-1D). Magnetic resonance imaging of the whole spine revealed intraspinal extradural mass extending from T4 until T7 level with the involvement of T4-T7 vertebral bodies, compressing onto the thecal sac. There were also enhancing prevertebral and right paravertebral lesions extending from T2 to T9 levels. The right pleura was thickened and enhancing with the presence of right pleural effusion (Figures 2A-2F).

Subsequently, a pleuroscopy was performed, and about 1.3 L of cloudy fluid was aspirated from the right pleural space and pleural biopsy was sent for histopathological evaluation. A chest tube was then inserted for drainage of the pleural effusion. Cytological examination of the pleural fluid revealed lymphocytosis with no malignant cell seen. The pleural fluid also showed an elevated glucose level of 6.1 mmol/L and protein level of 43 g/L. Histopathological examination of the pleural biopsy showed only chronic inflammation.

**Figure 3:**
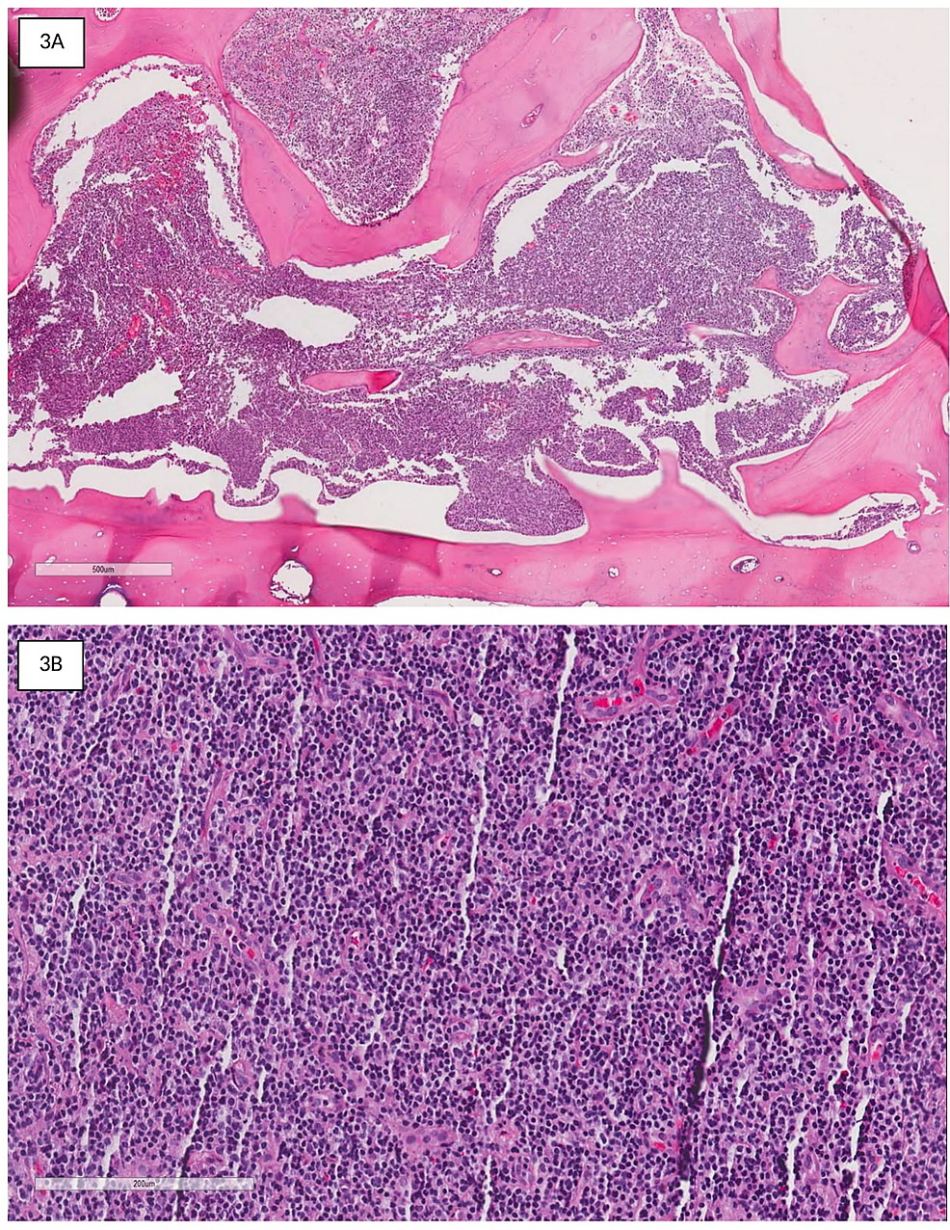
Histopathological examination revealed hypercellular marrow spaces infiltrated by malignant lymphoid cells (A: H&E 50x magnification). At 200x magnification, the malignant cells have round to oval vesicular nuclei. Some cells resemble immunoblast, centroblast, and centrocytes (B).

**Figure 4:**
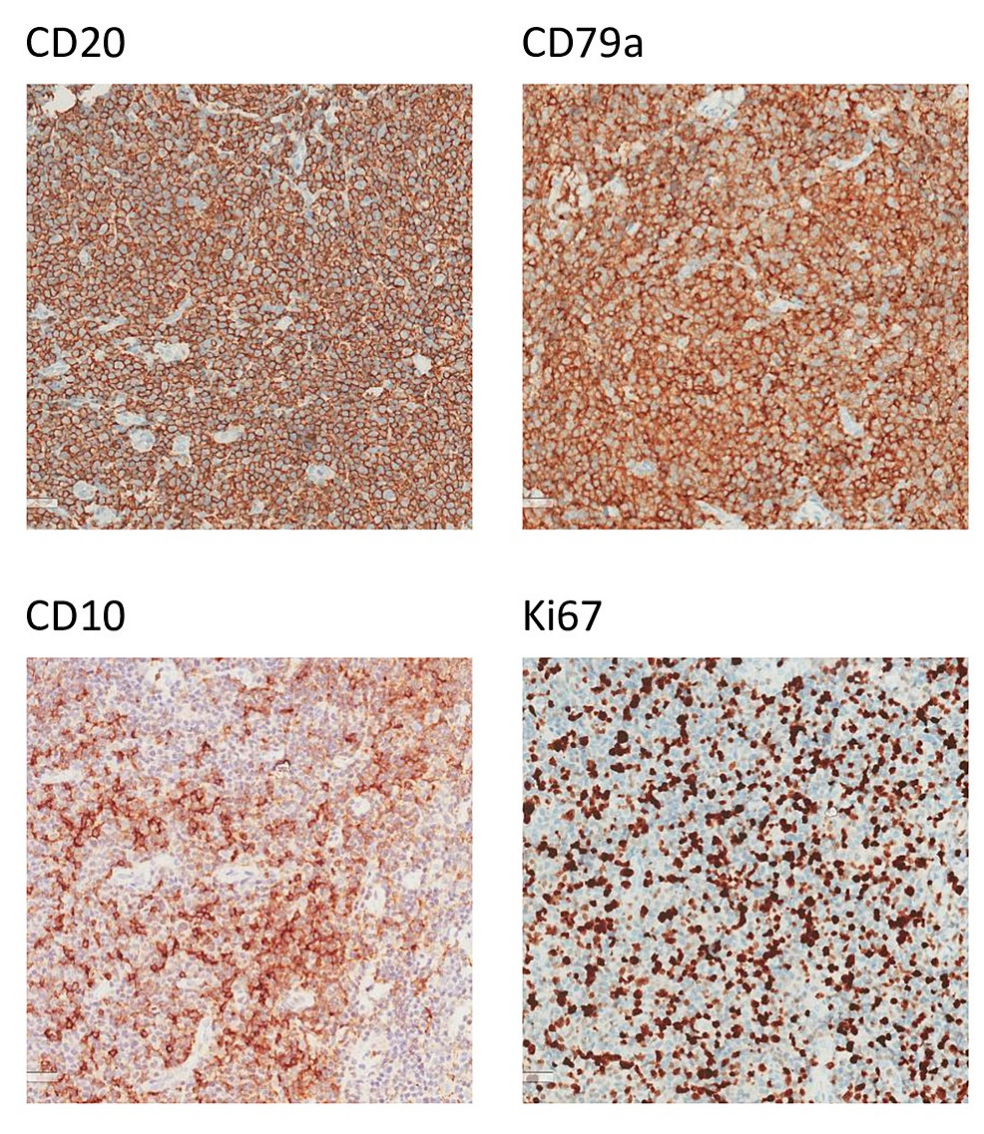
The malignant cells are positive for CD20, CD79a, and CD10. Ki67 proliferation rate is about 60%. CD: cluster of differentiation

**Figure 5:**
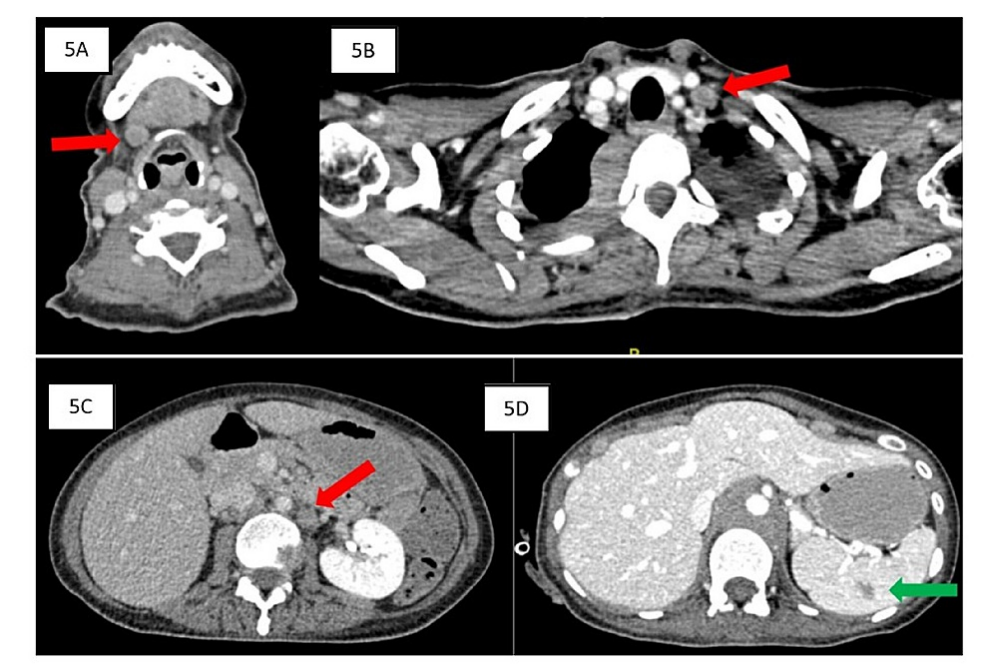
Contrast-enhanced CT images in axial sections. Multiple enlarged lymph nodes (red arrows) are seen at right submandibular (A), left supraclavicular (B), and para-aortic (C) regions. An ill-defined hypodense lesion at the spleen (D, green arrow) could represent lymphomatous infiltration.

Considering the long and insidious progress of symptoms combined with the aforementioned radiological findings, a provisional diagnosis of spinal tuberculosis was made. As the patient was not keen on further diagnostic procedures at that time, she was provisionally treated with antituberculosis agents. Nevertheless, despite after one month of antituberculosis chemotherapy, her clinical condition remained static without any improvement. After further counseling, she consented to diagnostic tissue biopsy and decompression with laminectomy from T3 until T7 level. Intra-operatively, there was an extradural mass compressing the spinal cord at T4-T7 level. However, the cord and dura appeared healthy. The extradural mass was brownish and soft with caseous material, and it was removed and sent for histopathological examination. A diagnosis of B-cell lymphoma was made based on histopathological findings (Figures 3A, 3B, 4). Staging with contrast-enhanced computed tomography revealed a stage IV disease with enlarged right submandibular, left supraclavicular, and left axillary lymph nodes as well as splenic and right pleural involvement (Figures 5A-5D). She was then referred to a hematologist for adjuvant chemo- and radiotherapy but she refused further treatment and she subsequently succumbed to the disease.

## Discussion

Tuberculosis (TB) is an endemic disease in Malaysia. It is estimated that 10% of the disease in Malaysia are extra-pulmonary TB and 4% of pulmonary TB patients have extra-pulmonary involvement [8]. Also known as the great mimicker, the clinical picture of TB spondylitis is often similar to that of pyogenic infection and neoplasm. Furthermore, a review by Kumaran et al. demonstrates that features of TB spine in magnetic resonance (MR) images can mimic other spinal pathologies, such as pyogenic spondylitis, rheumatoid arthritis, metastasis, lymphoma, fracture, degenerative disc disease, Baastrup’s disease, and Rosai-Dorfman disease [9]. To make it even more complicated, the tuberculin skin test (Mantoux test) is not a reliable test to confirm a tuberculosis infection [10]. Only 86% of patients with active TB have positive Mantoux tests [10]. Hence, without obtaining a histopathological sample for examination, a definitive diagnosis is often obscured.

In our patient, the onset of the symptoms was insidious and progressive. Clinically, there was no sign to suggest malignancy. Coupled with MR images that showed intraspinal extradural mass with vertebral body involvement and multiple lesions which enhanced post-contrast, a provisional diagnosis of TB spine was made despite an equivocal Mantoux test. As the patient had a strong aversion towards surgery initially, we started a trial of anti-TB chemotherapy but to no avail. However, excision of the extradural lesion was successfully performed and histopathological examination of the mass finally revealed primary B cell lymphoma of the spine as the true culprit in this patient.

Primary non-Hodgkin lymphoma of the spine is a rare condition. In fact, a review of the literature reveals only case reports and case series, highlighting the rarity of this entity [5]. In contrast to our patient, the largest series presented by Tang et al. demonstrate that the onset of spinal NHL is normally acute [5]. This might be due to her delayed presentation to the hospital after symptom onset. Upon presentation, she has spinal cord compression which is consistent with all the patients with spinal NHL [5]. Soft tissue extension from the spinal NHL is the main cause of spinal cord compression [5]. 

In terms of radiological investigations, plain radiographs of the spine are usually non-specific and often tend to underestimate the extent of disease. MR imaging is the radiological examination of choice as it is non-invasive and it can clearly delineate the extension of lesion and help in the planning of surgical intervention. Spinal NHL can present as an iso- or hypo-intense lesion on T1-weighted images, hyperintense on T2-weighted images with a homogenous enhancement post-gadolinium administration [11]. In this patient, the MRI reveals an extradural lesion with possible intradural extension from T4 to T7 level with pleural thickening. These findings prompt us to include spinal tuberculosis as one of our differential diagnoses.

The gold standard in the diagnosis of spinal NHL is the histopathological examination (HPE). Biopsy can be obtained at the time of operative intervention or done as a separate procedure, either percutaneous or open. In cases where there is no indication for open surgery, CT-guided biopsy is certainly a viable alternative, especially if the lesion is in an extradural location.

Despite the advancement of chemo- and radiotherapies in managing patients with malignancy, primary spinal NHL seems to have an unfavorable prognosis [12]. The median life expectancy for an untreated patient with primary spinal NHL is three months. Poor prognostic factors include elderly age, aggressive histological types, paraplegia, bladder and bowel involvement, poor performance status, elevated LDH, and high protein in the cerebral spinal fluid as well as deep-seated tumor [12]. Furthermore, in lieu of the rarity of this disease, the optimal treatment for it remains a topic of debate [5]. Reports show that treatment usually consists of chemotherapy, radiotherapy, surgery, or a combination of these [5].

The chemotherapy of choice is the CHOP regime [5]. CHOP is named after the initials of the drugs used which include cyclophosphamide, doxorubicin (hydroxydaunomycin), vincristine (Oncovin®), and prednisolone. Another regime called R-CHOP, in which there is the inclusion of monoclonal antibody (rituximab), is also commonly used.

The goals of initial spinal surgery are usually to prevent further bone destruction and compression, to assist weight-bearing, to obtain pain relief and a better quality of life. But the ever-present dilemma in these cases is the fact that patients with malignant tumors are often elderly, debilitated, or systemically ill from their malignant diseases. Often, extensive operative procedures like laminectomy and posterior instrumentation carry more risks than benefits in this group of patients. In this patient, we have performed a decompressive laminectomy to alleviate the spinal cord compression, during which we are also able to excise the brownish caseous extradural mass for histopathological examination.

## Conclusions

In a conclusion, the diagnosis of primary NHL of the thoracolumbar spine is often missed or delayed due to a lack of specific findings and the presence of confusing similarities with other diseases. Even though the main form of treatment is chemotherapy and/or radiotherapy in cases of spinal lymphoma, surgery may still be indicated in patients who present with neurological symptoms. More importantly, HPE should be obtained to guide our treatment plans and for prognostication purposes. With regard to HPE, the soft tissue component of the lesion is preferred over bone, as inadequate tumor cells in biopsies taken from lytic areas of medullary infiltration have been reported.
